# Survival of Patients Diagnosed With Cancer During the COVID-19 Pandemic

**DOI:** 10.1001/jamaoncol.2025.6332

**Published:** 2026-02-05

**Authors:** Todd Burus, Haluk Damgacioglu, Bin Huang, Thomas C. Tucker, Ashish A. Deshmukh, Krystle A. Lang Kuhs

**Affiliations:** 1 Markey Cancer Center, University of Kentucky, Lexington; 2 Division of Biomedical Informatics, College of Medicine, University of Kentucky, Lexington; 3 Department of Public Health Sciences, Medical University of South Carolina, Charleston; 4 Hollings Cancer Center, Medical University of South Carolina, Charleston; 5 Division of Cancer Biostatistics, College of Medicine, University of Kentucky, Lexington; 6 Kentucky Cancer Registry, Markey Cancer Center, University of Kentucky, Lexington; 7 Department of Epidemiology & Environmental Health, College of Public Health, University of Kentucky, Lexington

## Abstract

**Importance:**

The effects of COVID-19 pandemic–related disruptions on cancer diagnosis in the US have been widely observed, but their impact on short-term survival has not been assessed.

**Objective:**

To examine 1-year cause-specific survival (CSS) rates among patients diagnosed with cancer during 2020 and 2021 using high-quality cancer registry data.

**Design, Setting, and Participants:**

This population-based cohort study of cancer survival between 2015 and 2021 used the Surveillance, Epidemiology, and End Results 21 Registries (SEER-21) database. The population included individuals with an invasive cancer diagnosis and complete follow-up reported to registries included in SEER-21 between January 1, 2015, and December 31, 2021. Data were analyzed from May 13 to May 27, 2025.

**Exposure:**

COVID-19 pandemic.

**Main Outcomes and Measures:**

The primary outcome was 1-year CSS rates by stage at diagnosis for patients diagnosed with cancer in 2020 and 2021 compared with trends in 1-year CSS rates among patients diagnosed between 2015 and 2019. Additional site-specific analyses were performed on common cancer sites identified as having low survival (5-year relative survival <33%) or high incidence and high survival (incidence >20.0 per 100 000 and 5-year relative survival ≥66%).

**Results:**

A total of 1 008 012 individuals were diagnosed with cancer during the first 2 years of the COVID-19 pandemic, including 473 781 in 2020 (50.1% female; 51.0% diagnosed at ≥65 years; 11.5% Black, 15.8% Hispanic, 64.1% White, and 7.4% other race) and 534 231 in 2021 (50.3% female; 51.9% diagnosed at ≥65 years; 51.9% diagnosed at ≥65 years; 11.7% Black, 16.2% Hispanic, 62.9% White, and 7.9% other race). Compared with prepandemic trends, significant absolute reductions in 1-year CSS rates occurred for early-stage diagnoses in 2020 (−0.44 [95% CI, −0.54 to −0.34] percentage points) and 2021 (−0.27 [95% CI, −0.37 to −0.16] percentage points) and late-stage diagnoses in 2020 (−1.34 [95% CI, −1.75 to −0.93] percentage points) and 2021 (−1.20 [95% CI, −1.69 to −0.71] percentage points). Survival reductions resulted in an estimated 17 390 more cancer-related deaths (13.1%) within 1 year of diagnosis than expected during the first 2 years of the COVID-19 pandemic. Absolute survival reductions greater than 1.00 percentage point occurred in both years for late-stage diagnoses among individuals of other non-Hispanic race and ethnicity (ie, American Indian and Alaska Native, Asian or Pacific Islander, or unknown race) and individuals aged 65 years or older. Significant site-specific survival reductions also existed in both 2020 and 2021, respectively, for early-stage diagnoses of esophageal cancer (−3.89 and −3.67 percentage points) and colorectal cancer (−1.08 and −0.78 percentage points) and late-stage diagnoses of prostate cancer (−0.64 and −0.77 percentage points).

**Conclusions and Relevance:**

This cohort study found that individuals diagnosed with cancer in 2020 and 2021 experienced worse short-term survival than those diagnosed between 2015 and 2019, suggesting substantial harms associated with cancer care disruptions during the first 2 years of the COVID-19 pandemic.

## Key Points

**Question:**

Were the survival rates of patients diagnosed with cancer in the US in 2020 and 2021 different than in the years before the COVID-19 pandemic?

**Findings:**

This population-based cohort study of more than 1 million US individuals found that patients diagnosed with cancer in 2020 and 2021 (compared with 2015 to 2019) had significant reductions in survival when diagnosed at both early and late stages, resulting in an estimated 17 390 more deaths (13.1%) within 1 year of diagnosis than expected.

**Meaning:**

Individuals diagnosed with cancer in the US during 2020 and 2021 experienced worse survival during the first year after their diagnosis than those diagnosed before the COVID-19 pandemic.

## Teaser

This cohort study investigates the association of the COVID-19 pandemic with short-term survival among patients diagnosed with cancer in 2020 and 2021 using US population-based cancer registry data.

## Introduction

Several studies have reported significant COVID-19 pandemic–related disruptions to cancer care in the US.^1,2,3,4,5,6,7,8,9,10^ Yet, with current cancer registry data covering only cases diagnosed through 2022, existing research has mostly been restricted to evaluating declines in cancer screening and diagnosis. An important and unstudied question is the association of these disruptions with increased cancer morbidity and mortality. While it will likely take several years to gain a complete picture of how the pandemic has impacted cancer outcomes, 1-year cause-specific survival (CSS) among patients diagnosed in 2020 and 2021 can be evaluated using available data. In this study, we analyzed 1-year CSS to determine whether—and to what extent—short-term cancer-related outcomes changed during the first 2 years of the COVID-19 pandemic.

## Methods

This study was deemed exempt from review by the University of Kentucky Institutional Review Board, and informed consent was waived because the data were deidentified and publicly available. We followed the Strengthening the Reporting of Observational Studies in Epidemiology (STROBE) reporting guidelines.

### Data Sources

We obtained 1-year CSS data for patients diagnosed with a first primary malignant cancer in the US between January 1, 2020, and December 31, 2021, using data from the Surveillance, Epidemiology, and End Results 21 Registries (SEER-21) database.^11^ SEER-21 covered approximately 41.9% of the US population during the study period. Cases in which the individual was diagnosed by death certificate and/or autopsy only, or recorded as alive with no survival time available, were excluded, as were in situ cases or cases with unknown staging. We collected data for the overall population and stratified by sex, age, race and ethnicity, urbanicity, and stage at diagnosis. Age was stratified at age 65 years or older to account for the beginning of Medicare eligibility and the fact that advanced age was a leading risk factor for COVID-19 mortality. Race and ethnicity were defined as Hispanic, non-Hispanic Black, non-Hispanic White, and other non-Hispanic races according to the SEER-recommended race and origin variable.^12^ Other non-Hispanic races included individuals who identified as American Indian and Alaska Native and Asian or Pacific Islander, which were combined due to low case counts. Urbanicity was defined by the 2013 Rural Urban Continuum Codes (RUCC) for county of residence, with RUCC 1-3 defined as metropolitan and RUCC 4-9 as nonmetropolitan.^13^ Stage at diagnosis was defined using SEER’s combined summary stage variable with expanded regional codes, with early stage defined as localized disease only, and late stage including regional and distant stage diagnoses.^11^

We also collected separate CSS data for individual cancer sites according to 2 mutually exclusive categories of interest, which we labeled low-survival cancers and high-incidence/high-survival cancers. Low-survival cancers are susceptible to survival outcome changes with even minor disruptions in care delivery, whereas small reductions in CSS for high-incidence/high-survival cancers could entail large increases in total cancer deaths due to the large number of total diagnoses. We defined low-survival cancers as common cancer sites with current 5-year relative survival rates of approximately one-third or lower.^14^ These sites included the pancreas, liver and intrahepatic bile duct, esophagus, lung and bronchus, and the brain and other nervous system sites. We defined high-incidence/high-survival cancers as common cancer sites with a current age-adjusted incidence greater than or equal to 20.0 per 100 000 people and a 5-year relative survival of approximately two-thirds or higher.^14^ These sites included the female breast, prostate, colon and rectum, corpus and uterus (not otherwise specified), and melanoma. Cancer sites were identified according to the *International Classification of Diseases for Oncology, Third Revision* Site Recode/World Health Organization 2008 definition.^15^

### Statistical Analysis

Data were analyzed from May 13 to May 27, 2025. We calculated 1-year CSS rates overall and by stage at diagnosis with 95% CIs for patients diagnosed with malignant cancer in 2020 and 2021 using SEER*Stat, version 8.4.5 (National Cancer Institute).^16^ Annual 1-year CSS rates were also calculated for 2015 to 2019 for comparison. CSS rates were calculated among the total eligible population using the actuarial method (eMethods in Supplement 1). One-year CSS estimates the probability of not having a cancer-related cause of death within 1 year of diagnosis and is calculated as the number of persons still living 1 year after a cancer diagnosis divided by the total number of persons diagnosed with cancer. CSS is a measure of net cancer survival in which noncancer causes of death are censored. We chose CSS for this analysis instead of relative survival due to concerns over having appropriate life tables for computing the expected survival of individuals in the absence of a cancer diagnosis during the COVID-19 pandemic.^17,18,19^

Previous research has indicated substantial differences in the number of cancer cases diagnosed between 2020 and 2021.^2^ The increase in 2021 may be attributed, in part, to the diagnosis of cases that went undiagnosed in 2020. Therefore, to account for potential changes in stage distribution during the COVID-19 pandemic, we analyzed survival trends separately by stage at diagnosis. Stage-specific 1-year CSS trends for 2015 to 2019 were fit with proportional hazards models using the JPSurv web application.^20^ Fitted models estimated trends as the average absolute change in survival (AACS) with 95% CIs. AACS represents the absolute year-to-year percentage point difference in 1-year CSS between 2015 and 2019. The fitted models for 2015 to 2019 were then used to estimate expected stage-specific 1-year CSS rates for 2020 and 2021 and to calculate the absolute survival difference between the observed and expected 1-year CSS rates. Additionally, we calculated the 1-year mortality difference as the difference in the number of observed and expected deaths within 1 year of diagnosis. Stage-specific 1-year mortality differences were obtained by multiplying the absolute survival difference by the appropriate observed cancer incidence rate and the standard US population estimate of 274 633 642 (or 137 316 821 for sex-specific cancers). Total 1-year mortality differences in 2020 and 2021 were calculated as the sum of stage-specific 1-year mortality differences. Because CSS is not an additive measure, the 1-year mortality difference for all cancer sites will not necessarily equal the sum of 1-year mortality differences among complementary subgroups.

We defined pointwise significant changes in CSS to have occurred in 2020 or 2021 if the 95% CI for the absolute difference between observed and expected 1-year CSS rates did not contain 0. All analyses were performed and visualizations created using the R statistical programming language, version 4.4.1 (R Foundation).

## Results

### One-Year Cause-Specific Survival for All Cancer Sites

During the first 2 years of the COVID-19 pandemic, a total of 1 008 012 individuals were diagnosed with cancer, with 473 781 cases (47.0%) reported in 2020 (50.1% female and 49.9% male; 51.0% diagnosed at ≥65 years; 11.5% Black, 15.8% Hispanic, 64.1% White, and 7.4% other race, including Alaska Native, American Indian, Asian, and Pacific Islander) and 534 231 cases (53.0%) in 2021 (50.3% female and 49.7% male; 51.9% diagnosed at ≥65 years; 11.7% Black, 16.2% Hispanic, 62.9% White, and 7.9% other race, including Alaska Native, American Indian, Asian, and Pacific Islander) (Table 1). Among these, 143 577 individuals experienced a cancer-related death within 1 year of diagnosis (69 779 in 2020 and 73 798 in 2021), and 63 967 were lost to follow-up (26 784 in 2020 and 37 183 in 2021). The resulting 1-year CSS rates were 84.84% in 2020 (95% CI, 84.74%-84.95%) and 85.69% in 2021 (95% CI, 85.59%-85.78%). Total case numbers by site and stage at diagnosis are included in eTables 1 and 2 in Supplement 1.

**Table 1.** Cancer Diagnoses and Deaths Within 1 Year of Diagnosis Among Patients in SEER-21

Group	Patients, No. (%)					
	2015-2019		2020		2021	
	Cases	Deaths	Cases	Deaths	Cases	Deaths
Overall No.	2 471 372	366 968	473 781	69 779	534 231	73 798
Stage at diagnosis						
Early stage	1 216 058 (49.2)	42 978 (11.7)	228 692 (48.3)	8487 (12.2)	266 540 (49.9)	9195 (12.5)
Late stage	1 255 314 (50.8)	323 990 (88.3)	245 089 (51.7)	61 292 (87.8)	267 691 (50.1)	64 603 (87.5)
Age group, y						
<65	1 252 085 (50.7)	124 814 (34.0)	232 146 (49.0)	22 813 (32.7)	256 885 (48.1)	23 249 (31.5)
≥65	1 219 287 (49.3)	242 154 (66.0)	241 635 (51.0)	46 966 (67.3)	277 346 (51.9)	50 549 (68.5)
Race and ethnicity						
Hispanic	367 991 (14.9)	52 329 (14.3)	74 759 (15.8)	10 747 (15.4)	86 411 (16.2)	11 969 (16.2)
Non-Hispanic Black	280 365 (11.3)	45 354 (12.4)	54 320 (11.5)	8655 (12.4)	62 523 (11.7)	9166 (12.4)
Non-Hispanic White	1 623 286 (65.7)	243 755 (66.4)	303 836 (64.1)	45 112 (64.6)	335 769 (62.9)	46 876 (63.5)
Other non-Hispanic^a^	177 959 (7.2)	24 495 (6.7)	35 271 (7.4)	4982 (7.1)	42 266 (7.9)	5532 (7.5)
Unknown	21 771 (0.9)	1035 (0.3)	5595 (1.2)	283 (0.4)	7262 (1.4)	255 (0.3)
Urbanicity						
Metropolitan	2 174 826 (88.0)	313 505 (85.4)	416 680 (87.9)	59 463 (85.2)	471 631 (88.3)	62 932 (85.3)
Nonmetropolitan	294 426 (11.9)	52 988 (14.4)	56 691 (12.0)	10 218 (14.6)	62 136 (11.6)	10 763 (14.6)
Unknown	2120 (0.1)	475 (0.1)	410 (0.1)	98 (0.1)	464 (0.1)	103 (0.1)

Abbreviation: SEER-21, Surveillance, Epidemiology, and End Results 21 Registries.

^a^ Other non-Hispanic races included individuals who identified as American Indian and Alaska Native and Asian or Pacific Islander.

**Figure 1.**
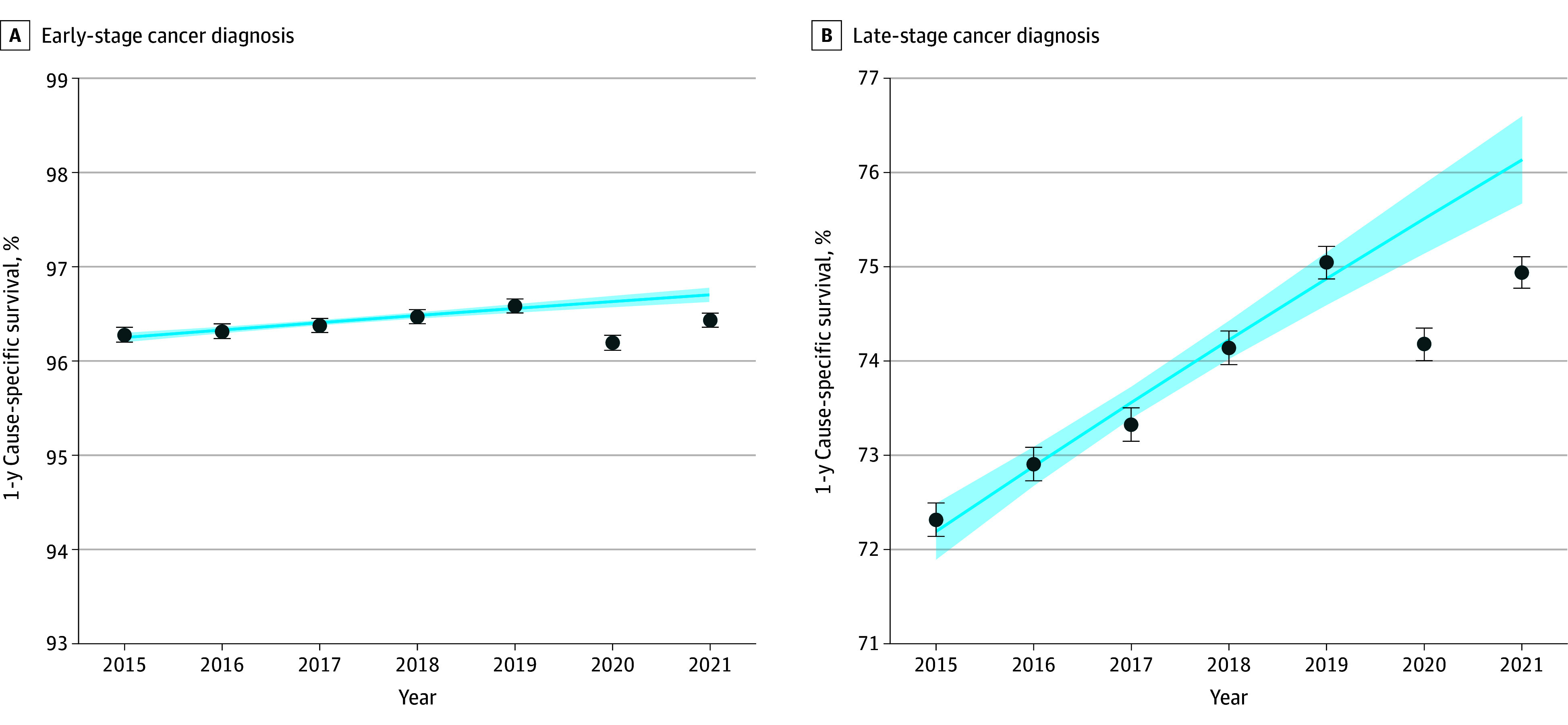
Changes in 1-Year Cause-Specific Survival (CSS) Rates Among Patients Diagnosed With Cancer in 2020 and 2021 One-year CSS rates with 95% CIs among patients diagnosed with early-stage cancer (A)and late-stage cancer (B) between 2015 and 2021. Shaded areas represent 95% CIs. The overall number of patients at risk was 1 008 012.

**Figure 2.**
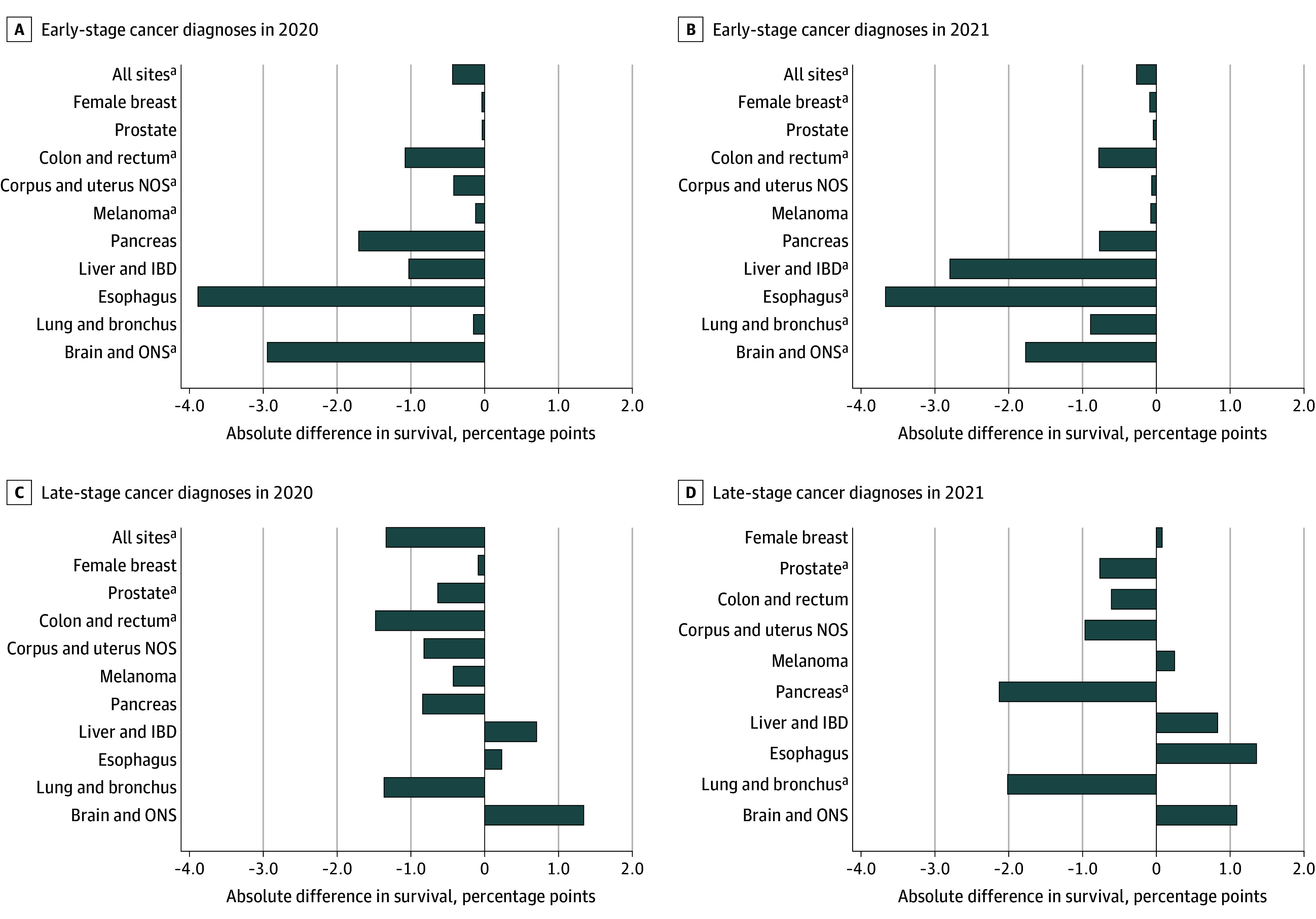
Changes in 1-Year Cause-Specific Survival (CSS) Rates Among Patients Diagnosed With Cancer in 2020 and 2021 by Cancer Site Absolute differences in 1-year CSS rates among patients with early-stage cancer diagnoses in 2020 (A), early-stage cancer diagnoses in 2021 (B), late-stage cancer diagnoses in 2020 (C), and late-stage cancer diagnoses in 2021 (D). The overall number of patients are risk was 473 781 in 2020 and 534 231 in 2021. IBD indicates intrahepatic bile duct; NOS, not otherwise specified; ONS, other nervous system. ^a^Significant difference from expected.

In 2020, there were 228 692 early-stage cancer diagnoses, with a corresponding 1-year CSS rate of 96.20% (95% CI, 96.11%-96.28%) (Table 2; Figure 1). This number increased to 266 540 early-stage diagnoses in 2021, with a 1-year CSS of 96.43% (95% CI, 96.36%-96.51%). Compared with survival trends from 2015 to 2019, the 1-year CSS for early-stage diagnoses was 0.44 percentage points lower than expected in 2020 (95% CI, −0.54 to −0.34 percentage points) and 0.27 percentage points lower in 2021 (95% CI, −0.37 to −0.16 percentage points) (Figure 2).

**Table 2.** Absolute Difference in Observed and Expected 1-Year Cause-Specific Survival (CSS) Rates for Patients Diagnosed With Cancer in 2020 and 2021 by Group and Stage at Diagnosis in SEER-21

Group and stage at diagnosis	2020		2021	
	Observed CSS, % (95% CI)	Difference from expected, percentage points (95% CI)	Observed CSS, % (95% CI)	Difference from expected, percentage points (95% CI)
**Overall**				
Early stage	96.20 (96.11 to 96.28)	−0.44 (−0.54 to −0.34)	96.43 (96.36 to 96.51)	−0.27 (−0.37 to −0.16)
Late stage	74.18 (74.00 to 74.35)	−1.34 (−1.75 to −0.93)	74.94 (74.77 to 75.10)	−1.20 (−1.69 to −0.71)
**Age group, y**				
<65				
Early stage	98.08 (98.00 to 98.16)	−0.25 (−0.37 to −0.13)	98.24 (98.17 to 98.31)	−0.14 (−0.27 to −0.01)
Late stage	82.01 (81.79 to 82.23)	−0.92 (−1.59 to −0.25)	82.80 (82.58 to 83.01)	−0.64 (−1.46 to 0.17)
≥65				
Early stage	94.29 (94.15 to 94.43)	−0.66 (−0.85 to −0.48)	94.69 (94.57 to 94.81)	−0.41 (−0.61 to −0.21)
Late stage	66.83 (66.57 to 67.10)	−1.77 (−2.14 to −1.40)	67.86 (67.61 to 68.11)	−1.59 (−2.00 to −1.18)
**Race and ethnicity**				
Hispanic				
Early stage	95.81 (95.59 to 96.02)	−0.21 (−0.54 to 0.12)	95.92 (95.72 to 96.12)	−0.13 (−0.50 to 0.24)
Late stage	76.23 (75.81 to 76.65)	−1.42 (−2.24 to −0.61)	76.45 (76.05 to 76.84)	−1.69 (−2.65 to −0.73)
Non-Hispanic Black				
Early stage	96.34 (96.11 to 96.58)	−0.47 (−0.73 to −0.22)	96.75 (96.55 to 96.95)	−0.12 (−0.36 to 0.12)
Late stage	72.42 (71.90 to 72.94)	−1.18 (−1.90 to −0.45)	73.31 (72.81 to 73.80)	−0.94 (−1.73 to −0.14)
Non-Hispanic White				
Early stage	96.14 (96.05 to 96.24)	−0.50 (−0.62 to −0.39)	96.38 (96.29 to 96.47)	−0.35 (−0.47 to −0.23)
Late stage	73.53 (73.31 to 73.76)	−1.24 (−1.69 to −0.80)	74.33 (74.12 to 74.55)	−1.05 (−1.58 to −0.52)
Other non-Hispanic^a^				
Early stage	96.61 (96.33 to 96.90)	−0.27 (−0.81 to 0.27)	96.69 (96.43 to 96.94)	−0.19 (−0.83 to 0.45)
Late stage	76.51 (75.91 to 77.12)	−2.09 (−2.82 to −1.36)	77.31 (76.75 to 77.87)	−2.01 (−2.77 to −1.25)
**Urbanicity**				
Urban				
Early stage	96.34 (96.26 to 96.43)	−0.39 (−0.48 to −0.30)	96.61 (96.54 to 96.69)	−0.19 (−0.27 to −0.10)
Late stage	74.83 (74.64 to 75.01)	−1.30 (−1.79 to −0.82)	75.61 (75.43 to 75.79)	−1.14 (−1.73 to −0.56)
Rural				
Early stage	95.05 (94.79 to 95.32)	−0.79 (−1.18 to −0.40)	95.05 (94.80 to 95.30)	−0.92 (−1.34 to −0.49)
Late stage	69.67 (69.15 to 70.20)	−1.46 (−2.11 to −0.81)	70.15 (69.65 to 70.66)	−1.59 (−2.28 to −0.89)

Abbreviation: SEER-21, Surveillance, Epidemiology, and End Results 21 Registries.

^a^ Other non-Hispanic races included individuals who identified as American Indian and Alaska Native and Asian or Pacific Islander.

For late-stage diagnoses, 245 089 cases were identified in 2020 with a 1-year CSS of 74.78% (95% CI, 74.00%-74.35%), while 267 691 late-stage cases were recorded in 2021 with a 1-year CSS of 74.94% (95% CI, 74.77%-75.10%). In both years, 1-year CSS for late-stage cases was significantly lower than expected based on prepandemic survival trends, with 1.34 percentage points lower in 2020 (95% CI, −1.75 to −0.93 percentage points) and 1.20 percentage points lower in 2021 (95% CI, −1.69 to −0.71 percentage points).

Reductions in survival among both early-stage and late-stage cancers contributed to an estimated mortality difference of 9162 (95% CI, 6958-11 365) cancer-related deaths within 1 year of diagnosis in 2020 and 8228 (95% CI, 5411-11 044) in 2021 (Table 3). Overall, the first 2 years of the pandemic were associated with 17 390 more cancer-related deaths (13.1%) than expected within 1 year of diagnosis.

**Table 3.** One-Year Mortality Difference by Cancer Site in SEER-21

Site	Mortality difference, No. (95% CI)^a^	
	2020	2021
All sites	9162 (6958 to 11 365)	8228 (5411 to 11 044)
Female breast	88 (−273 to 448)	60 (−415 to 536)
Prostate	249 (38 to 461)	346 (71 to 621)
Colon and rectum	1150 (704 to 1595)	645 (88 to 1202)
Corpus and uterus NOS	189 (−79 to 457)	130 (−217 to 477)
Melanoma	81 (−34 to 196)	15 (−107 to 137)
Pancreas	333 (23 to 644)	652 (298 to 1006)
Liver and IBD	31 (−359 to 421)	231 (−285 to 747)
Esophagus	70 (−105 to 245)	−16 (−218 to 186)
Lung and bronchus	1131 (−5 to 2268)	1966 (538 to 3393)
Brain and ONS	341 (148 to 533)	197 (−4 to 397)

Abbreviations: IBD, intrahepatic bile duct; NOS, not otherwise specified; ONS, other nervous system sites; SEER-21, Surveillance, Epidemiology, and End Results 21 Registries.

^a^ One-year mortality difference is defined as the difference in the number of observed and expected deaths due to cancer within 1 year of diagnosis. Expected deaths in 2020 and 2021 are based on trends in 1-year cause-specific survival between 2015 and 2019 and the observed cancer incidence rates in 2020 and 2021.

All population subgroups examined experienced 1-year CSS reductions for early-stage diagnoses in both years, except for non-Hispanic Black individuals in 2021 and individuals of Hispanic ethnicity and other non-Hispanic race in 2020 and 2021 (Table 2). Late-stage survival reductions occurred in all population subgroups in 2020 and in all except individuals younger than 65 years in 2021. Absolute survival reductions of greater than 1.00 percentage points occurred in both years for late-stage diagnoses among individuals of other non-Hispanic race (−2.09 [95% CI, −2.82 to −1.36] percentage points and −2.01 [95% CI, −2.77 to −1.25] percentage points, respectively) and individuals aged 65 years and older (−1.77 [95% CI, −2.14 to −1.40] percentage points and −1.59 [95% CI, −2.00 to −1.18], respectively).

### One-Year Cause-Specific Survival for Low-Survival Cancers

The 5 low-survival cancer sites considered accounted for 17.8% of all cases included in 2020 and 2021 and 52.3% of all cancer deaths within 1 year of diagnosis. Significant reductions in 1-year CSS for early-stage diagnoses occurred in 2020 for esophageal cancer (−3.89 percentage points; 95% CI, −6.78 to −0.99 percentage points) and brain cancer (−2.94 percentage points; 95% CI, −4.29 to −1.60 percentage points), and in 2021 for liver cancer (−2.80 percentage points; 95% CI, −5.14 to −0.45 percentage points), esophageal cancer (−3.67 percentage points; 95% CI, −6.33 to −1.01 percentage points), lung cancer (−0.89 percentage points; 95% CI, −1.59 to −0.19 percentage points), and brain cancer (−1.77 percentage points; 95% CI, −3.17 to −0.37 percentage points) (Figure 2; eTable 3 in Supplement 1). Survival reductions were also observed in 2021 for late-stage diagnoses of pancreatic cancer (−2.13 percentage points; 95% CI, −3.16 to −1.09 percentage points) and lung cancer (−2.01 percentage points; 95% CI, −3.74 to −0.30 percentage points). Overall, significant 1-year mortality differences were observed for brain cancer in 2020 (341 more deaths than expected; 95% CI, 98-148), lung cancer in 2021 (1966 more deaths than expected; 95% CI, 538-3393), and pancreatic cancer in both years (333 and 652 more deaths than expected; 95% CIs, 23-644 and 298-1006).

### One-Year Cause-Specific Survival for High-Incidence/High-Survival Cancers

The 5 high-incidence/high-survival cancer sites considered accounted for 44.0% of all cases reported in 2020 and 2021 and 14.0% of all cancer deaths within 1 year of diagnosis. In 2020, 1-year CSS rates were significantly lower than expected for early-stage colorectal cancer (−1.08 percentage points; 95% CI, −1.55 to −0.61 percentage points), uterine cancer (−0.42 percentage points; 95% CI, −0.74 to −0.10 percentage points) and melanoma (−0.12 percentage points; 95% CI, −0.24 to −0.01 percentage points) and for late-stage prostate cancer (−0.64 percentage points; 95% CI, −1.25 to −0.03 percentage points) and colorectal cancer (−1.48 percentage points; 95% CI, −2.24 to −0.72 percentage points). One-year CSS rates were significantly lower than expected in 2021 for early-stage female breast cancer (−0.09 percentage points; 95% CI, −0.17 to −0.04 percentage points), early-stage colorectal cancer (−0.78 percentage points; 95% CI, −1.29 to −0.26 percentage points) and late-stage prostate cancer (−0.77 percentage points; 95% CI, −1.45 to −0.08 percentage points). Individuals diagnosed with prostate cancer and colorectal cancer experienced significantly more deaths than expected within 1 year of diagnosis in both 2020 and 2021, resulting in an estimated 25.4% more prostate cancer deaths and 16.3% more colorectal cancer deaths than would have occurred without survival reductions.

## Discussion

To our knowledge, this population-based cohort study is the first to examine short-term survival outcomes among US patients with newly diagnosed cancers during the first 2 years of the COVID-19 pandemic. We found significant reductions in 1-year CSS rates among patients diagnosed with both early-stage and late-stage cancer in 2020 and 2021 compared with trends from 2015 to 2019. Overall, survival reductions in 2020 and 2021 resulted in an estimated 17 390 more cancer deaths (13.1%) than expected within the first 12 months after diagnosis. Reductions occurred among all considered population subgroups, with the greatest impact among individuals of other non-Hispanic race and ethnicity and individuals aged 65 years or older. We also observed site-specific decreases among a variety of low-survival and high-incidence/high-survival cancer sites. We found the results for colorectal, prostate, and pancreatic cancers most concerning because all experienced significant differences in 1-year mortality in 2020 and 2021 after accounting for differences in stage distribution. Overall, our findings indicate that individuals diagnosed with cancer in the US during 2020 and 2021 experienced poorer cancer-related outcomes in the first year after diagnosis than those diagnosed before the COVID-19 pandemic.

Previously, Mani et al^17^ reported that persons living with cancer were at a greater risk of death from COVID-19 infection in 2020 than those without a cancer diagnosis, even at less than 1 year from diagnosis. Using cancer-specific mortality for our study allowed us to minimize concerns about COVID-19–related mortality and focus attention on experiences along the cancer care continuum. Given that available treatments should have been no worse than before the pandemic, decreases in 1-year CSS for patients diagnosed in 2020 and 2021 suggest issues with timely diagnosis and treatment.^21,22,23,24^ Our findings of significant 1-year survival rate reductions overall and for many of the cancer sites examined support this hypothesis and align with prior research into potential increased mortality due to treatment delays during the COVID-19 pandemic.^25^

Considering specific sites as either low-survival or high-incidence/high-survival allowed us to examine CSS in 2020 and 2021 from different perspectives. It was not surprising to find that several cancer sites with traditionally low survival rates experienced greater-than-expected 1-year mortality associated with pandemic-related disruptions during 2020 and 2021. It was somewhat surprising, however, that with just 1 year of follow-up, we already detected significantly lower CSS for several high-incidence/high-survival cancer sites as well. While the magnitude of reductions for these sites was generally less than observed among low-survival cancer sites, their higher incidence rates translated to a substantial increase in estimated lives lost vs expected. Underdetection of less advanced or asymptomatic cases during 2020 and 2021 may help explain some of the reductions observed in short-term survival for high-incidence/high-survival cancer sites. However, accounting for stage at diagnosis in our final assessment mitigated this concern to some extent. Moreover, even if some of these results are due to underdetection, this is not very reassuring because undetected cases could have progressed further before being discovered—something that may already be reflected in the survival rates from 2021. Identifying policy missteps and failures in health system preparedness that may have contributed to underdetection of cancer—as well as the previously mentioned delays in time to treatment initiation and treatment disruptions—are fundamental issues remaining to be addressed in the wake of the COVID-19 pandemic. It is important to investigate these questions further and determine whether other explanations exist as to why high-survival cancer sites were vulnerable to poorer outcomes during the COVID-19 pandemic.

Reductions in cancer screening leading to a change in the composition of cancers diagnosed in 2020 and 2021 are also important to consider when discussing survival decreases. Though some studies reported reduced female breast cancer screening during 2020, Doan et al^26^ found that screening disruptions among Medicare enrollees were largely relegated to the initial pandemic months of March through May. Additionally, prior studies from our group found that breast cancer incidence returned to expected levels by June 2020.^1,2^ These observations may help explain the limited 1-year survival impact we witnessed for female breast cancer. In contrast, prolonged screening disruptions and corresponding estimates of underdiagnosis for all stages of colorectal and lung cancers are consistent with our findings of reduced stage-specific survival for these sites.^1,2,3,4,5,6,27,28^ Disruptions to lung cancer screening have been attributed to COVID-19 infection concerns and strained health care capacity.^5,21,26,29,30^ Similar concerns were also raised regarding a lower volume of colonoscopies performed in 2020.^6,31^ As such, rebuilding capacity and reminding patients about their eligible cancer screenings will be critical to limiting the long-term negative impacts of the COVID-19 pandemic on early cancer detection. Rebuilding capacity and taking other steps to instill health system resilience would also be effective measures to help prevent delays in time to treatment initiation and treatment disruptions for patients with cancer in general.^24,32,33,34^

### Strengths and Limitations

A key strength of this study is its use of data from high-quality, population-based central cancer registries. However, we acknowledge certain limitations within this study. First, using relative survival instead of CSS may be preferred for measuring excess mortality at the specific sites we examined,^35,36^ but restricting to only 1 year of follow-up limits the differences that can emerge. Moreover, previous studies have shown that CSS is preferable if standard life tables are unavailable or fail to capture risk factors specific to patients with cancer, such as increased risk of COVID-19 mortality.^17,18,19,37^ Second, differences in the severity of cases diagnosed may exist and affect survival between the pandemic and prepandemic periods. Stratifying survival rates by stage at diagnosis helps mitigate this concern, though meaningful within-stage variations may exist beyond the scope of this study to assess. Further research is needed to clarify this difference and better prepare for future disruptions. Third, CSS estimates may change due to delays in reporting deaths for patients diagnosed in 2020 and 2021, though this effect is likely minimal given that the dataset used was not assembled until 22 months after the latest follow-up date. Fourth, patient history of nonfatal COVID-19 infection or other type of infection was not available, limiting our ability to consider how an earlier recovered SARS-CoV-2 infection could have led to worse overall frailty in a way that it would not have done in a patient without cancer. Fifth, although we restricted consideration to cancer-specific causes of death, the accuracy of cause-of-death information during the COVID-19 pandemic may be reduced due to misclassification of COVID-19–related mortality. However, in an additional analysis, we found larger reductions in relative survival (which does not depend on cause of death) than those found for CSS (eTable 4 in Supplement 1). This pattern indicates misclassification is unlikely to be a primary driver of reductions in CSS, although some misclassification may still be present. Prior literature also supports using SEER cause-of-death fields for estimating cancer-specific mortality despite some misclassification.^19,37,38,39^

## Conclusions

Disruptions related to the COVID-19 pandemic in the US have had a substantial impact across the cancer care continuum. This cohort study’s findings of significantly decreased rates of short-term cancer survival during the pandemic’s first 2 years are in line with previously documented interruptions to screening, diagnosis, and treatment. Survival reductions were seen in a broad range of cancer sites and stages of diagnosis, regardless of available screenings or typical prognosis. Swift action should be taken to increase cancer screening, rebuild health care capacity, and improve patient communication to combat longer-term consequences and prepare for potential future disruptions. Continued surveillance is needed to assess whether additional changes in survival outcomes extended further into and after the pandemic.

## Notes

Supplement 1. **eMethods.** One-Year Cause-Specific Survival **eTable 1.** Number of Cancer Cases Diagnosed at an Early Stage by Site and Time Period **eTable 2.** Number of Cancer Cases Diagnosed at a Late Stage by Site and Time Period **eTable 3.** Absolute Difference In Observed and Expected 1-Year Cause-Specific Survival (CSS) Rates for Patients Diagnosed With Cancer in 2020 and 2021 by Site and Stage at Diagnosis **eTable 4.** Comparison of Changes in 1-Year Cause-Specific Survival (CSS) Rates Versus 1-Year Relative Survival (RS) Rates

Supplement 2.
Data Sharing Statement
