# Supplementary material for: Survival of Patients Diagnosed With Cancer During the COVID-19 Pandemic
Source: JAMA Oncol. 2026 Feb 5;12(4):356–63. doi: 10.1001/jamaoncol.2025.6332 (PMC12878639; doi:10.1001/jamaoncol.2025.6332)
Supplement: Supplement 1. — eMethods. One-Year Cause-Specific Survival eTable 1. Number of Cancer Cases Diagnosed at an Early Stage by Site and Time Period eTable 2. Number of Cancer Cases Diagnosed at a Late Stage by Site and Time Period eTable 3. Absolute Difference In Observed and Expected 1-Year Cause-Specific Survival (CSS) Rates for Patients Diagnosed With Cancer in 2020 and 2021 by Site and Stage at Diagnosis eTable 4. Comparison of Changes in 1-Year Cause-Specific Survival (CSS) Rates Versus 1-Year Relative Survival (RS) Rates [file jamaoncol-e256332-s001.pdf]

## Supplemental Online Content

Burus T, Damgacioglu H, Huang B, et al. Survival of patients diagnosed with cancer during the COVID-19 pandemic. *JAMA Oncol*. Published online February 5, 2026. doi:10.1001/jamaoncol.2025.6332

**eMethods.** One-Year Cause-Specific Survival

**eTable 1.** Number of Cancer Cases Diagnosed at an Early Stage by Site and Time Period

**eTable 2.** Number of Cancer Cases Diagnosed at a Late Stage by Site and Time Period

**eTable 3.** Absolute Difference In Observed and Expected 1-Year Cause-Specific Survival (CSS) Rates for Patients Diagnosed With Cancer in 2020 and 2021 by Site and Stage at Diagnosis

**eTable 4.** Comparison of Changes in 1-Year Cause-Specific Survival (CSS) Rates Versus 1-Year Relative Survival (RS) Rates

**eReferences**

This supplemental material has been provided by the authors to give readers additional information about their work.

© 2026 Burus T et al. JAMA Oncology.

**eMethods. One-year cause-specific survival.**

Cause-specific survival (CSS) is a measure of net survival for a specific disease or cause of death; deaths from other causes are censored. One-year CSS estimates the probability of surviving the cause of death of interest for one year post-diagnosis by measuring the total number of individuals still alive with the disease one year after diagnosis divided by the number of individuals alive with the disease at the start of the study period. Individuals with other causes of death are censored and considered as "lost to follow-up" for calculations, as are individuals whose survival status at one year following diagnosis is unknown. The Actuarial Method can be applied to adjust calculations for individuals lost to follow-up.

The process for calculating one-year CSS using the Actuarial Method is as follows:

- $\mathbf{A}$ = the number of individuals alive with the disease at the start of the study period
- $\mathbf{D}$ = the number of individuals that die of the disease within one-year of diagnosis
- $\mathbf{U}$ = the number of individuals lost to follow-up.
- To account for individuals lost to follow-up, calculate an adjusted alive value $\mathbf{A}^* = \mathbf{A} - \frac{\mathbf{1}}{\mathbf{2}}\mathbf{U}.$
- Given $\mathbf{A^*}$ and $\mathbf{D}$, then we can calculate one-year CSS, $\mathbf{P} = \mathbf{1} - \mathbf{D}/\mathbf{A}^*$

When using SEER*Stat to estimate cause-specific survival, cause of death is determined using the SEER cause-specific death classification. The SEER cause-specific death classification system provides a detailed algorithm for determining cancer-attributable causes of death based on *International Classification of Diseases* codes.[1]

© 2026 Burus T et al. JAMA Oncology.

**eTable 1. Number of cancer cases diagnosed at an early stage by site and time period, Surveillance, Epidemiology, and End Results Program, 21 Registries[2]**

| | Cases, n (%) | | |
|---|---|---|---|
| **Site** | **2015-2019** | **2020** | **2021** |
| Female Breast | 263389 (21.66) | 49408 (21.60) | 58671 (22.01) |
| Prostate | 265223 (21.81) | 52312 (22.87) | 62503 (23.45) |
| Colon and Rectum | 80251 (6.60) | 13547 (5.92) | 15858 (5.95) |
| Corpus and Uterus NOS | 66428 (5.46) | 12836 (5.61) | 14492 (5.44) |
| Melanoma | 89264 (7.34) | 15569 (6.81) | 18944 (7.11) |
| Pancreas | 10683 (0.88) | 2561 (1.12) | 2987 (1.12) |
| Liver and IBD | 28680 (2.36) | 5194 (2.27) | 5864 (2.20) |
| Esophagus | 4534 (0.37) | 913 (0.40) | 1065 (0.40) |
| Lung and Bronchus | 62620 (5.15) | 11830 (5.17) | 13484 (5.06) |
| Brain and ONS | 29457 (2.42) | 5920 (2.59) | 6037 (2.26) |
| Other Cancer Sites | 315529 (25.94) | 58602 (25.62) | 66635 (25.00) |

Abbreviations: NOS = not otherwise specified; IBD = intrahepatic bile duct; ONS = other nervous system

© 2026 Burus T et al. JAMA Oncology.

**eTable 2. Number of cancer cases diagnosed at a late stage by site and time period, Surveillance, Epidemiology, and End Results Program, 21 Registries[2]**

| | Cases, n (%) | | |
|---|---|---|---|
| **Site** | **2015-2019** | **2020** | **2021** |
| Female Breast | 139686 (11.13) | 26983 (11.01) | 29769 (11.12) |
| Prostate | 81707 (6.51) | 17213 (7.02) | 20298 (7.58) |
| Colon and Rectum | 134781 (10.74) | 26352 (10.75) | 29550 (11.04) |
| Corpus and Uterus NOS | 27933 (2.23) | 5958 (2.43) | 6513 (2.43) |
| Melanoma | 16143 (1.29) | 3143 (1.28) | 3678 (1.37) |
| Pancreas | 61701 (4.92) | 12844 (5.24) | 13431 (5.02) |
| Liver and IBD | 27367 (2.18) | 5741 (2.34) | 6251 (2.34) |
| Esophagus | 17475 (1.39) | 3552 (1.45) | 3734 (1.39) |
| Lung and Bronchus | 206600 (16.46) | 36701 (14.97) | 38793 (14.49) |
| Brain and ONS | 5913 (0.47) | 1264 (0.52) | 1276 (0.48) |
| Other Cancer Sites | 536008 (42.69) | 105338 (42.98) | 114398 (42.73) |

Abbreviations: NOS = not otherwise specified; IBD = intrahepatic bile duct; ONS = other nervous system

© 2026 Burus T et al. JAMA Oncology.

**eTable 3. Absolute difference in observed and expected one-year cause-specific survival (CSS) rates for patients diagnosed with cancer in 2020 and 2021 by site and stage at diagnosis, Surveillance, Epidemiology, and End Results Program, 21 Registries**[2]

| | 2020 | | | 2021 | | |
|---|---|---|---|---|---|---|
| **Site** | **Observed (%, 95% CI)** | **Expected (%, 95% CI)** | **Difference (95% CI)** | **Observed (%, 95% CI)** | **Expected (%, 95% CI)** | **Difference (95% CI)** |
| Early Stage | | | | | | |
| Female Breast | 99.61 (99.55-99.66) | 99.65 (99.59-99.7) | -0.04 (-0.12 to 0.04) | 99.58 (99.53-99.63) | 99.67 (99.60-99.74) | -0.09 (-0.17 to 0.00) |
| Prostate | 99.77 (99.73-99.81) | 99.8 (99.77-99.84) | -0.04 (-0.09 to 0.02) | 99.78 (99.74-99.81) | 99.82 (99.78-99.85) | -0.04 (-0.09 to 0.01) |
| Colon and Rectum | 96.28 (95.95-96.60) | 97.35 (97.01-97.69) | -1.08 (-1.55 to -0.61) | 96.62 (96.33-96.91) | 97.4 (96.97-97.82) | -0.78 (-1.29 to -0.26) |
| Corpus and Uterus NOS | 98.53 (98.32-98.74) | 98.95 (98.70-99.19) | -0.42 (-0.74 to -0.10) | 98.92 (98.75-99.09) | 98.98 (98.68-99.29) | -0.06 (-0.41 to 0.29) |
| Melanoma | 99.61 (99.51-99.71) | 99.74 (99.67-99.80) | -0.12 (-0.24 to -0.01) | 99.68 (99.60-99.76) | 99.76 (99.68-99.83) | -0.08 (-0.19 to 0.03) |
| Pancreas | 66.14 (64.28-68.00) | 67.85 (65.99-69.70) | -1.71 (-4.34 to 0.92) | 67.56 (65.84-69.27) | 68.33 (65.95-70.70) | -0.77 (-3.70 to 2.16) |
| Liver and IBD | 76.43 (75.25-77.61) | 77.46 (75.83-79.08) | -1.03 (-3.03 to 0.98) | 74.97 (73.83-76.10) | 77.77 (75.71-79.82) | -2.8 (-5.14 to -0.45) |
| Esophagus | 75.09 (72.21-77.96) | 78.97 (78.61-79.33) | -3.88 (-6.78 to -0.99) | 75.82 (73.19-78.45) | 79.49 (79.04-79.94) | -3.67 (-6.33 to -1.01) |
| Lung and Bronchus | 91.86 (91.36-92.36) | 92.01 (91.60-92.42) | -0.15 (-0.80 to 0.50) | 91.52 (91.04-92.00) | 92.41 (91.91-92.92) | -0.89 (-1.59 to -0.19) |
| Brain and ONS | 65.46 (64.23-66.69) | 68.41 (67.85-68.96) | -2.94 (-4.29 to -1.60) | 66.69 (65.48-67.90) | 68.46 (67.76-69.17) | -1.77 (-3.17 to -0.37) |
| Late Stage | | | | | | |
| Female Breast | 93.27 (92.97-93.57) | 93.36 (92.81-93.91) | -0.09 (-0.72 to 0.54) | 93.36 (93.08-93.65) | 93.28 (92.57-94.00) | 0.08 (-0.69 to 0.85) |
| Prostate | 93.09 (92.71-93.47) | 93.73 (93.25-94.20) | -0.64 (-1.25 to -0.03) | 93.14 (92.79-93.49) | 93.91 (93.32-94.50) | -0.77 (-1.45 to -0.08) |
| Colon and Rectum | 79.25 (78.75-79.74) | 80.73 (80.15-81.30) | -1.48 (-2.24 to -0.72) | 80.39 (79.93-80.85) | 81 (80.28-81.73) | -0.61 (-1.47 to 0.25) |
| Corpus and Uterus NOS | 77.68 (76.61-78.75) | 78.5 (76.42-80.59) | -0.82 (-3.17 to 1.52) | 77.61 (76.58-78.64) | 78.58 (75.92-81.24) | -0.97 (-3.82 to 1.88) |
| Melanoma | 83.19 (81.87-84.52) | 83.62 (83.26-83.98) | -0.43 (-1.80 to 0.95) | 83.95 (82.75-85.16) | 83.71 (83.24-84.17) | 0.25 (-1.04 to 1.54) |
| Pancreas | 38.08 (37.23-38.93) | 38.92 (38.44-39.40) | -0.84 (-1.82 to 0.14) | 37.46 (36.62-38.29) | 39.58 (38.97-40.20) | -2.13 (-3.16 to -1.09) |
| Liver and IBD | 36.15 (34.88-37.42) | 35.45 (32.72-38.18) | 0.7 (-2.31 to 3.71) | 36.29 (35.07-37.51) | 35.46 (31.97-38.96) | 0.83 (-2.87 to 4.53) |
| Esophagus | 50.3 (48.63-51.97) | 50.07 (48.55-51.58) | 0.23 (-2.02 to 2.48) | 51.58 (49.95-53.21) | 50.22 (48.29-52.16) | 1.36 (-1.17 to 3.88) |
| Lung and Bronchus | 49.3 (48.77-49.82) | 50.66 (49.35-51.96) | -1.36 (-2.77 to 0.04) | 49.92 (49.41-50.43) | 51.93 (50.29-53.57) | -2.02 (-3.74 to -0.30) |
| Brain and ONS | 48.96 (46.16-51.76) | 47.62 (46.03-49.20) | 1.34 (-1.88 to 4.56) | 47.78 (44.98-50.57) | 46.68 (44.65-48.72) | 1.09 (-2.37 to 4.55) |

Abbreviations: CI = confidence interval; NOS = not otherwise specified; IBD = intrahepatic bile duct; ONS = other nervous system

© 2026 Burus T et al. JAMA Oncology.

**eTable 4. Comparison of changes in one-year cause-specific survival (CSS) rates versus one-year relative survival (RS) rates, Surveillance, Epidemiology, and End Results Program, 21 Registries.**[2]
Comparison of the difference between observed survival rates and expected survival rates based off trends from 2015 to 2019. Difference represents the absolute difference in percentage points with 95% confidence intervals (CI).

| | **2020** | | **2021** | |
|---|---|---|---|---|
| **Site** | **CSS Difference (%, 95% CI)** | **RS Difference (%, 95% CI)** | **CSS Difference (%, 95% CI)** | **RS Difference (%, 95% CI)** |
| Early Stage | | | | |
| All Cancer Sites | -0.44 (-0.54 to -0.34) | -0.48 (-0.59 to -0.36) | -0.27 (-0.37 to -0.16) | -0.14 (-0.25 to -0.02) |
| Colon and Rectum | -1.08 (-1.55 to -0.61) | -1.76 (-2.35 to -1.18) | -0.78 (-1.29 to -0.26) | -1.05 (-1.66 to -0.45) |
| Lung and Bronchus | -0.15 (-0.80 to 0.50) | -0.37 (-1.23 to -0.49) | -0.89 (-1.59 to -0.19) | -1.32 (-2.25 to -0.39) |
| Late Stage | | | | |
| All Cancer Sites | -1.34 (-1.75 to -0.93) | -1.79 (-2.16 to -1.42) | -1.20 (-1.69 to -0.71) | -1.56 (-2.00 to -1.13) |
| Colon and Rectum | -1.48 (-2.24 to -0.72) | -1.94 (-2.85 to -1.03) | -0.61 (-1.47 to 0.25) | -0.85 (-1.90 to -0.21) |
| Lung and Bronchus | -1.36 (-2.77 to 0.04) | -2.12 (-3.46 to -0.79) | -2.02 (-3.74 to -0.30) | -2.76 (-4.40 to -1.13) |

© 2026 Burus T et al. JAMA Oncology.

**eReferences**

1. National Cancer Institute. SEER Cause-specific Death Classification. National Cancer Institute: Surveillance, Epidemiology, and End Results Program. Accessed November 14, 2025. https://seer.cancer.gov/causespecific/

2. National Cancer Institute, DCCPS, Surveillance Research Program. Surveillance, Epidemiology, and End Results (SEER) Program (www.seer.cancer.gov) SEER*Stat Database: Incidence - SEER Research Plus Limited-Field Data, 21 Registries (excl IL), Nov 2024 Sub (2000-2022) - Linked To County Attributes - Total U.S., 1969-2023 Counties. Published online April 2025.

© 2026 Burus T et al. JAMA Oncology.
