# Supplementary material for: Survival of Patients Diagnosed With Cancer During the COVID-19 Pandemic
Source: JAMA Oncol. 2026 Feb 5;12(4):356–63. doi: 10.1001/jamaoncol.2025.6332 (PMC12878639; doi:10.1001/jamaoncol.2025.6332)
Supplement: Supplement 2. — Data Sharing Statement [file jamaoncol-e256332-s002.pdf]

Data Sharing Statement

Burus. Survival of Patients Diagnosed With Cancer During the COVID-19 Pandemic. *JAMA Oncol*. Published February 05, 2026. doi:10.1001/jamaoncol.2025.6332

Data
Data available: No

Additional Information
Explanation for why data not available: Data is available through a restricted use agreement with the National Cancer Institute.
